# Modern Treatment of Cholera Infantum

**Published:** 1904-09

**Authors:** Robert M. Sterrett

**Affiliations:** New York City, Former Demonstrator of Surgical Pathology, College Physicians and Surgeons, Chicago; Former Attending Physician, Westside Free Dispensary, Medical Department, Chicago


### MODERN TREATMENT OF CHOLERA INFANTUM.

BY ROBERT M. STERRETT, M.D., NEW YORK CITY,
Former Demonstrator of Surgical Pathology, College Physicians and Surgeons, Chicago; Former Attending Physician, Westside Free Dispensary, Medical Department, Chicago.

The importance of prompt and efficient treatment in cases of cholera infantum will not be questioned by any one who has been called to these little sufferers with their glassy eyes, pinched, blue skin, the corners of the little mouths that ought to be curves of infantile beauty, drawn down with the aspect of old age, the cold perspiration standing out upon the skin, vomiting even cold water, and the bowels running off with a watery, foul discharge.

That the heat of the summer months, by its depressing effect upon the nervous system even in adults, is an etiologic factor in cholera infantum, is beyond doubt.

That excess of unsuitable, partly fermented food is another, can be stated as true. The stomach becomes overloaded, the bowels distend with gases from excessive fermentation, the bacilli swarm throughout the intestinal tract and vomiting and diarrhea are only an expression of Nature's disapproval of, and an attempt to, remedy the condition.

There is rapid internal congestion—stomach, bowels and more to be dreaded, the brain. The skin shows, in consequence of the congestion within, marked contraction of the capillaries. There is pain in the abdomen, temperature of 102° to 105°, and small, weak accelerated pulse.

You all know the picture—what are you doing for its relief? Do you keep on giving the old astringents to lock up the poisonous bacilli-infected mass of waste material in the intestinal tract? Do you give some combination containing the deadly paregoric? If you are, there is a better, safer, more efficient way. Do not take my word for it, but if you do not get better results your way, look into this:

In the first place—something has to be done, and done quickly, if some of these cases are to be saved. If the temperature is high there is most likely to be threatened convulsions if they are not already present.

Atropin and aconitin are the two remedies for these conditions, and must be given promptly and frequently enough to produce the effect desired; the restoration of circulatory equilibrium. I usually dissolve a granule of each—atropin, gr. 1-250; aconitin, gr. 1-134, for each year of the child's age, plus one granule for waste, into 24 teaspoonfuls of water, right out of the teakettle (sterilized), and give a teaspoonful of the solution every 15 to 30 minutes until the convulsions are relieved and the reddening skin shows a return of blood to the capillaries. If the child is two years old, three granules each of the remedies; if four years old, five granules, etc.

Then, the intestinal tract, where the fight is really going on, must be cleaned out and kept clean. A dose of castor oil, or calomel—the latter in small ($\frac{1}{6}$ grain) doses, repeated every hour, or, preferred, in larger doses. Then in order to counteract the toxins already in large quantities in the intestinal tract and poisoning the blood, I use the sulphocarbolates of lime, sodium, or zinc, as the best, safest, most efficient intestinal antiseptics I know of. If there is severe diarrhea, I give the baby a granule, gr. $\frac{1}{6}$, of zinc sulphocarbolate dissolved in aconitin and atropin solution, and continue the sulphocarbolate alone every half-hour or hour, after the former two remedies have been discontinued—which is as soon as the temperature has been restored to near the normal and the convulsions have ceased.

This has nothing to do with any expectant treatment—expect the patient will die, but hope he will not; it means "something doing" from the very start, and it means, also, success in a very large per cent. of cases seen in time to put the method into operation. I am not going to give any personal statistics showing how

I save all, or nearly all of my cholera infantum cases; that is not of such practical use to others. But, the active principles with their certainty of action, do accomplish safely, pleasantly and quickly, results that the galenics never have; and, from the very fact of their uncertain alkaloidal strength, never can produce. And the rendering the *prima via* as near aseptic as possible by the use of the sulphocarbolates is one of the most important, far-reaching achievements of modern therapeutics.

To Prof. Waugh belongs the credit of bringing this fact before the profession. He experimented with these salts and found that, when pure, they can be "pushed to effect" in any case; adults receiving in typhoid fever, for example, as high as 60 to 100 grains in twenty-four hours. This combination (sulphocarbolates of lime, sodium and zinc) is, in my hands, an ideal preparation.

In cases where there is more irritation of a gastric nature, I prefer the sodium salt; when the diarrhea is very bad, the zinc salt, as mentioned before; in cases where there is great wasting, indicating great perversion of general nutrition, I use the sulphocarbolates of lime. Of course, the diet should be restricted to even sterilized water, or barley water, the first twenty-four hours. The mother must be convinced that this is necessary and that her child will not die from starvation, in so short a time, but will be better for not adding any more fermentable material to the alimentary canal.

After the fight is over, strychnin arsenate, or brucin, in young babies, is the best tonic to restore and maintain vitality, we possess; they may be given as were the aconitin and atropin.

Finally, the cause of the attack is usually in and around the premises; these should be cleaned up and disinfected thoroughly, from cellar to garret, and the ejecta deodorized and sterilized, before being relegated to the sewer.

Proper guarded feeding, and avoidance of direct rays from the sun and the overcrowding of living and sleeping apartments, fresh air and pure water are all necessary adjuncts to any modern remedial measures and we all know how important they are. The active principle method of administering drugs is not so universally appreciated but, even in the conservative East, where they are exceedingly slow to "catch on" sometimes, the reasonableness and practicability of this direct, efficient advance in therapeutic procedure, is gaining very rapidly.—*St. Louis Courier of Medicine.*

